# Donor reliance and the impact on neglected tropical disease programme delivery: reflections and solutions for change from programme management perspectives

**DOI:** 10.1093/inthealth/ihaa090

**Published:** 2020-11-05

**Authors:** Karsor Kollie, Alice Siakeh, Georgina Zawolo, Anna Wickenden, Sally Theobald, Emerson Rogers, Zeela Zaizay, Rachael Thomson, Laura Dean

**Affiliations:** Neglected Tropical Disease Programme, Ministry of Health, Government of Liberia, Monrovia, Monsterrado, Liberia; University of Liberia Pacific Institute for Research and Evaluation, Monrovia, Montserrado, Liberia; University of Liberia Pacific Institute for Research and Evaluation, Monrovia, Montserrado, Liberia; Centre for Neglected Tropical Diseases, Liverpool School of Tropical Medicine, Pembroke Place, Liverpool L3 5QA, UK; Centre for Neglected Tropical Diseases, Liverpool School of Tropical Medicine, Pembroke Place, Liverpool L3 5QA, UK; Neglected Tropical Disease Programme, Ministry of Health, Government of Liberia, Monrovia, Monsterrado, Liberia; Actions Transforming Lives (ACT), Monrovia, Monsteraddo, Liberia; Centre for Neglected Tropical Diseases, Liverpool School of Tropical Medicine, Pembroke Place, Liverpool L3 5QA, UK; Centre for Neglected Tropical Diseases, Liverpool School of Tropical Medicine, Pembroke Place, Liverpool L3 5QA, UK

**Keywords:** collaboration, integration, neglected tropical diseases, ownership partnership, programme planning

## Abstract

Health systems within many developing countries are reliant on donor funding and non-governmental development organisations (NGDOs); this has had positive results but also presents challenges to sustainability and national ownership, with national programmes needing to respond to changing donor priorities. Simultaneously, the WHO neglected tropical disease (NTD) roadmap 2021–2030 calls for increased country ownership and domestically financed NTD programmes. Focusing on Liberia and blending primary research from the COU**NTD**OWN consortium and personal programmatic experience, this commentary reflects on the sustainability and financing of NTD programme delivery within the current context. We explore the successes and challenges of current models of collaboration and opportunities to improve country ownership and sustainability.

## Introduction

The Liberian health sector has faced multiple shocks including conflict, Ebola and the ongoing COVID-19 pandemic. Consequently, the health system is reliant on donor and non-governmental development organisation (NGDO) financial and technical support.^1^ The Liberian Ministry of Health (MoH) reports that partners provided over US$30 000 000 of support to the sector from 2008 to 2012.^2^ The Liberian neglected tropical disease (NTD) programme manages all priority NTDs using an integrated approach, coordinating inputs from multiple donors and NGDO partners. This brings opportunities, successes and challenges that are important to reflect on. This commentary draws on primary research from the COU**NTD**OWN consortium (https://countdown.lstmed.ac.uk/) as well as programmatic experience and personal reflections from NTD programme staff. Specifically, we focus on three key learnings: (1) collective action between NTD programme managers, donors and NGDO partners is critical to ensure a sustainable future for the NTD programme in Liberia; (2) country ownership and leadership is essential and often influenced by the ability of national actors to shape programme and policy reform and direct funding flows; and (3) we will achieve more sustained progress toward combatting NTDs together than we will confined to disease silos and competing interests. These learnings are considered in relation to two key areas: (1) cross- and intra-sectoral collaboration and renumeration challenges; and (2) international partnerships, funding flows and limited direct government financial support. This commentary aims to suggest solutions to ongoing challenges to support strengthening of the health system and country ownership of the NTD programme in Liberia. We recognise and appreciate the pivotal role and support from all funding and NGDO partners in Liberia, and hope to work collaboratively with them to address the challenges identified and replicate and scale up positive practices to support ongoing improvement efforts in the health system.

## Cross- and intra-sectoral collaboration and renumeration challenges

Cross and intra-sectoral collaboration, integration and coordination of vertical programme delivery within broader health system structures is essential to ensure that scarce resources are used effectively to strengthen health systems in low-income countries.^3^ This was emphasised by key informants who participated in implementation research conducted by the COU**NTD**OWN consortium^4^ and supported by the experiences of the NTD programme team (KK, ER and ZZ):

*If you have a scarce resource and you integrate them it is believed that you are going to use less resources to get maximum output. So, we assumed that integrating will help us use the scarce resources to have a large output* (COU**NTD**OWN key informant, MoH staff member, national level).

There are positive practices in coordination, collaboration and integration across the health system in Liberia that have led to positive programme outcomes. For example, by drawing on key approaches such as annual, integrated, multi-partner programme planning meetings that encourage collective budgeting as well as advocating for resource sharing between programmes at the county level (e.g. logistics for supervision), we have been able to address implementation funding gaps and ensure that the majority of programmatic activities receive financial support. Furthermore, international collaboration meetings through the Mano River Union have strengthened cross-border activities. Finally, the integration of case management programmes and the provision of cross-departmental technical assistance through working group meetings have meant that siloed approaches have become more harmonised.^4^ We attribute these collective efforts to improvements in the preventive chemotherapy coverage index in Liberia during 2017 (71%) and 2018 (77%).^5^

However, despite these successes, bottlenecks in effective coordination and collaboration remain. For example, at the national level, we found collaboration between departments within the MoH, as well as other sectors, is usually very cordial but sometimes compromised due to competing priorities and demands for attendees to receive remuneration, particularly when collaboration activities are perceived as donor-funded.^4^ To alleviate such challenges, a shift in thinking among governance actors and programme-implementing partners is critical. Remuneration to attend routine meetings that support cross-sectoral and inter-sectoral collaboration is unlikely and unsustainable. Thus, to promote better links, alternative motivation approaches should be explored. For example, participatory cross-departmental agenda-setting processes that may support this shift could be explored. The focus of these workshops could also be to work with all stakeholders to emphasise the benefits of inter-sectoral collaboration as a new approach to increase resource utilisation, enhance health service effectiveness and access to care.^3,6^

Programme interventions require skilled and knowledgeable personnel, and it should be acknowledged during planning and budgeting that the most committed individuals need to be compensated for the work that they do. In conflict-affected and fragile states, the government may not have the financial capacity to fulfil the health workforce payroll and this can lead to gross staff salary inadequacy and frequent payment delays.^7^ This not only restricts intra- and cross-sector collaboration, it also leads to high staff turnover and weak retention of national staff within the MoH.^8^ To mitigate this, some NGDOs provide performance-based top-up incentives for health professionals. Examples of such performance-based incentives include monthly top-up incentives provided by the Global Fund to TB, HIV and malaria MoH staff.^9^ Some NGDOs provide other means that can supplement income; others may not due to policy reasons. Some NGDO partners often argue that provision of financial incentives to MoH staff would take away ownership and make the government complacent, whereas others have identified that top-up cash incentives could be used to support the government to retain and attract skilled professionals where they are experiencing funding deficits and may also deter corruption.^10^ There is not a simple solution, but remuneration is an issue that needs to be carefully considered as we move towards a new sustainable model of partnership and domestic resource mobilisation.

## International partnerships, funding flows and limited direct government financial support

The Liberian NTD programme, like the entire health system, currently depends on donor and NGDO financial support to implement its activities. The restriction of these funds and erratic funding flows may not hinder effective vertical programme delivery in the short term, but based on personal experience (KK) and as reflected in the COU**NTD**OWN data,^4^ this does present challenges to government ownership and integrated programme delivery as described within the new WHO NTDs roadmap for 2021 to 2030.^11^

*Some partners could have a vested interest in a specific strategy and not actually in line with Government policy or what the Government wants to do…There could be a partner that thinks that we should just do onchocerciasis…we need to do an elimination by 2025, so why do we want to integrate with the others that you have a target of 2030?* (COU**NTD**OWN key informant, MoH staff member, county level).

NGDOs are sometimes unable to fully support government-planned interventions due to resource limitations or alternative policy priorities. For example, funds intended for disease-specific NTD training (e.g. onchocerciasis) may not be used for integrated NTDs training (e.g. training to include lymphoedema management). To maximise the impact and effectiveness of funds, NGDOs and government stakeholders should support joint priority integrated planning, recognising the competing priorities that need to be negotiated, particularly in the context of scarce government resources.

Strengthening both government leadership and ownership requires working with existing approaches and systems, and the relinquishing of some control of national policies by partners.^7^ We recommend increasing interaction of national programme managers and policymakers including donors to jointly negotiate programme plans, prioritise resource allocation, set milestones and develop interventions that are responsive to national priorities at all levels of service delivery. For example, the development and implementation of the Strategic Plan for Integrated Case Management of NTDs in Liberia is a model we advocate for. Unlike other prescribed documents and strategies, this is the first of its kind, led and owned by the country, with full interaction and engagement of all key partners including service providers across all levels of the health system and community representatives. Resource mobilisation for the implementation of this plan demands coordination and flexibility among NGDO partners and resource allocation is prioritised based on milestones set by the NTD programme. Following the first year of this integrated approach in five supported counties, the diagnosis of target NTDs (Buruli ulcer, leprosy, yaws, lymphedema and hydrocele) increased significantly; for example, in one county (Maryland), the number of health system-reported cases of targeted NTDs rose from 44 to 237 (a 439% increase).^12^

Technical and financial support from NGDO partners is critical, however, increased financial contributions from the national government are also essential to promote a shift towards local ownership.^11^ We have strived to seek the national government’s support by appointing a National NTDs Ambassador who has strong social and political connections and has guided strategic programme engagement with parliament and the finance ministry. These efforts have resulted in direct, yet limited and fluctuating, governmental financial inputs into NTD programme interventions. Without sustained and direct government support and responsive funding flows, true autonomy and sustainability in programme delivery are unlikely to be realised.

## The way forward

Support from donors and NGDO partners has been pivotal to the gains made in the NTD programme in Liberia and, as we move towards the ‘endgame’ for some NTDs, we must promote collective action between NTD programme managers, donors and NGDO partners to address the issues identified and to replicate positive practices. This will ensure a sustainable future for the NTD programme in Liberia. We have learned from years of programme experience and primary research that strengthening the health system requires country ownership and leadership. It is our experience that through evolving current partnerships using a positive, accountable and flexible approach, we will achieve much more together than we will confined to silos and competing interests.

## Data Availability

All relevant data is included within the manuscript.
